# Harnessing the Power of Difference: Colonialism and British Chronic Disease Research, 1940–1975

**DOI:** 10.1093/shm/hkv130

**Published:** 2015-11-12

**Authors:** Martin D. Moore

**Keywords:** public health, chronic disease epidemiology, postcolonial medicine, tropical medicine

## Abstract

Recent studies of post-war chronic disease epidemiology have generally focused on the histories of research in the USA and UK. Using the archival records of a major British funding body, the Colonial Medical Research Committee and its successor the Tropical Medical Research Board, this article demonstrates the advantages of bringing a post-colonial analytic to this historiography. It highlights how the administrative and medical interests in population difference at the centre of the new epidemiology came to map onto political apparatus initially created to know, reform and govern colonial subjects. Although detached from imperial aims, British medical scientists nonetheless attached value to colonial populations on the basis of British benefit and turned various sites into laboratories to extract it. This relationship did not die with the end of imperial rule. British scientists continued to pursue chronic disease epidemiology in former colonies well into the post-war period, informing debates about Britain's own public health concerns.

In 1964, the social medicine academic Jerry Morris published a greatly expanded second edition of his renowned book, *The Uses of Epidemiology.* As Morris noted in an earlier preface for the work, a key aim for the text was thinking through how novel techniques in epidemiology might be deployed to confront the apparent increase of non-infectious disease mortality in the middle aged, and in particular in middle-aged men.^1^ Foreshadowing current-day discourse, Morris conceived of mortality from conditions like coronary heart disease (CHD) in this population as a ‘modern epidemic’, one that could not be stemmed by the sanitary and environmental interventions inherited from the nineteenth century.^2^ Instead, building on previous work, Morris advocated identifying the ‘ways of living’ that underpinned the rising tide of mortality, and using this knowledge to alter them ‘without having to scrap western civilisation’.^3^

Yet, as Morris admitted, his conception of chronic diseases as resulting from numerous culturally inflected personal behaviours, held its own challenges, as well as promises. In particular, he felt future work would need to untangle the links between the many apparent causes involved, and to understand the effects of behaviours ‘so common and so widespread, so highly interrelated and all pervasive in modern industrial societies' that they could rarely be isolated, either conceptually or effectively, in single studies.^4^

One method that Morris felt offered hope for generating hypotheses was the comparative study of populations. For some purposes, a comparison between rural and urban locations within the same nation might suffice. However, he saw more powerful accounts as resulting from comparing populations living in more starkly divergent living conditions. Lacking the defining, and potentially pathological, features of modern society, communities ‘at an early stage of social and economic development’ supposedly provided opportunities for untangling webs of causation for various diseases.^5^ As Morris was very well aware, this was a programme which had formed the basis of substantial British research into CHD and related conditions during the decade prior to the publication of *Uses*. Moreover, it was an approach to ‘evidence-based’ chronic disease prevention that Morris himself would defend within high status research bodies into the decade after it.

Despite the interest that British epidemiologists began to pay to colonial populations in light of this new methodology, the interconnections between chronic disease research and colonialism have not been discussed in recent histories of chronic disease and risk-factor epidemiology.^6^ The expansion of epidemiological and public health interest into non-infectious disease has been well discussed, and in the British context Morris's work itself has provided a bellwether for broader trends.^7^ Nonetheless, historians have framed Britain's ‘New Public Health’ primarily in terms of the rise of European networks and of Anglo-American exchange, and they generally have not considered the manner in which Britain continued to be shaped by its imperial connections during and after decolonisation.^8^

Using a mixture of published material and the records of a major British funding body, the Colonial Medical Research Committee (CMRC, later the Tropical Medical Research Board), this article reasserts the importance of Britain's empire in Britain's own post-war encounter with chronic disease. Focusing on British research into a major public health concern—CHD, and predominantly the risk factor hypertension—it argues that the new epidemiological drive for studying cultural, social and biological difference led British researchers to turn colonial populations into research subjects. Whilst not inherently tied to imperial ambitions, Britain's deep colonial connections ensured that administrative and medical interests in population difference mapped onto a political apparatus created to know, reform and govern colonial subjects.^9^ It was an alignment predicated upon colonial rule's historical construction—and attempted elimination—of otherness.^10^

This article recognises, however, that the coloniality at the heart of chronic disease research was of a different kind to other colonial medical projects. Biomedicine here was not a tool of imperial exclusion, nor was it part of a civilising, modernising mission. Research was thus neither about refining methods of control, imposing new norms of behaviour, nor reshaping social and economic relations.^11^ Instead, researchers made use of colonial architectures—both during and after colonial rule—for the purpose of extracting lessons for disease prevention in Britain. Engagement, in this sense, was about domestic populations benefiting from the study of Britain's ‘othered’ populations in a manner far more direct than previous medical exercises.^12^ And benefits here were clearly linked to the emergence of new concerns in Britain itself.

Of course, the researchers and institutions of interest here were not bound solely by colonial and national state structures. As in earlier and alternative forms of colonial medicine, Britain's medical scientists moved smoothly in international circles. Similarly, both they and their research units received finance and support from transnational charitable bodies and health organisations.^13^

Yet, it is proposed here that the engagement of chronic disease researchers in such circles arose precisely because of their colonial and post-colonial experiences, whilst their engagement in colonial enterprises rested upon integration in prominent British research structures. This entanglement at various levels of research work gave the findings from colonial and post-colonial territories the means to impact upon British medicine in return. Nation and empire, that is, not only shaped each other to the point of interdependency, but together they also structured the mean flows of British interaction with international medicine.^14^

The remainder of this article will be divided into four sections. The first will outline the emergence of Britain's colonial research architecture, and the second the gradual inclusion of chronic disease research within its remit. Through a study of the founding and work of the Epidemiological Research Unit in Jamaica, the third section will then extend this history into the postcolonial period, and consider the entangled nature of its research with metropolitan problems and knowledge bases. The article will conclude by rearticulating the importance of postcolonial frames of investigation for future scholarship on British post-war public health and on international histories of chronic disease research.

## Creating Research Architecture in the Colonies

The two decades after 1940 witnessed a significant shift in the organisation and scale of medical research in the British Empire.^15^ Prior to this point, the colonies had provided space for much investigation into nutritional deficiencies, as well as for research into a host of communicable and vector-borne diseases central to the scientific speciality of tropical medicine.^16^ In terms of institutions, Britain's Indian and South Asian territories possessed a number of significant research centres, whilst academic and philanthropic bodies like the Liverpool School of Tropical Medicine and the Rockefeller Foundation had also established institutes in Caribbean and African colonies.^17^

In the period after 1940, however, colonial officials placed greater emphasis on colonial development in Africa and the Caribbean, with a concomitant effect on the way that the colonial state engaged with medical research. During the 1920s and 1930s, colonial officials had often framed development in terms of piecemeal infrastructure and public health works, undertaken to improve the production and transport of various cash crops and minerals for international export. In return, they hoped to generate demand for British manufactured goods and improve employment rates in Britain.^18^ After 1940, a broader view of development emerged, in light of not just wartime exigencies, but also violent colonial unrest and severe domestic and international criticism of empire.^19^ Through a series of Colonial Development and Welfare (CD&W) Acts, Colonial Office officials gave greater emphasis to the creation of education and social welfare structures in a more paternalist mode of governance.^20^ Although benefits to Britain were still expected, officials toned down discourses of British advantage. Instead, they emphasised Britain's role in ‘guid[ing] colonial people along the road to self-government’.^21^ And in economic terms, agriculture rather than industry dominated plans for the colonies, with civil servant and ministerial visions for agricultural improvement dominated by a high-modernist technocratic ethos. Development bodies thus produced a number of large-scale, state-led schemes, based around mechanisation and technical knowledge.^22^

As Sabine Clarke has pointed out, scientific research moved to the centre of this expanded dedication to colonial modernisation, operationalised through the CD&W Acts.^23^ Although scientific knowledge had previously been linked closely with the aim of colonial modernisation, the Acts went far and beyond the scope of previous Colonial Development legislation.^24^ By contrast to the £600,000 spent on research projects under the old Colonial Development Act of 1929, the new CD&W Act of 1940 specified £500,000 per year for research purposes, growing to £1 million p/a in 1945 and thereafter.^25^ Moreover, the end of the War also witnessed the creation of a number of University Colleges in African and Caribbean colonies—some with attatched medical schools—providing the institutional basis for a developing research culture.^26^

As a result of these Acts, the Colonial Office was placed in control of a substantial sum of research funding, and it sought a technocratic solution to allocating its budget and managing projects. Incorporating the expertise of eminent British scientists and research councils, the Office created a series of committees to advise on its research activities. For medicine, it established the Colonial Medical Research Committee in 1945 as a joint venture with the Medical Research Council (MRC).^27^ The political fortunes of the Committee have been discussed elsewhere.^28^ Suffice to say here, though, that through its fifteen years of existence, the Committee was given considerable autonomy to organise and allocate funding to applicants, albeit policy shifts towards devolution and indigenisation during the 1950s saw its freedom to act gradually curtailed.^29^ Nonetheless, through its continued policy of funding research units overseas, as well as providing delegates to new regional institutions, the CMRC played a prominent role in funding and overseeing research work in the colonies until 1960.^30^

## The Emergence of Non-infectious Diseases as Targets of Colonial Research

Between the Committee'e creation in 1945 and its reformulation in 1960, the vast majority of its funding was allocated to classic colonial and tropical medicine concerns.^31^ For instance, between 1950 and 1954, the CMRC allocated £572,559 from the CD&W funds for research and administrative purposes. Of this, £130,000 was spent on nutritional research at field stations, and £212,000 was spent on virus and vector-borne disease work, most prominently on malaria programmes (£79,000).^32^ Even where the techniques applied to research were novel—for instance, metabolic investigations of nutritional deficiency—the targets of research were consistent with earlier colonial interests.^33^ Furthermore, continuing an earlier tradition of colonial medicine, new overseas units and research programmes maintained close connections with institutions or researchers in the UK.^34^ Colonial territories remained the ‘field’ for UK researchers visiting to collect samples or data for analysis at ‘home’.^35^ Thus, while the Committee insisted that research should be driven by individual interests and performed where most practicable, changes in scale and organisation at a service level did not necessarily translate to a shift in the frameworks underlying colonial research.

One area in which the underpinning nature of research did change, however, was in terms of funding for non-infectious disease research. Although consuming only a minor proportion of total research expenditure towards the end of the Committee's lifetime, interest in incurable non-infectious diseases increased significantly in the second half of the 1950s.^36^

Initial curiosity and investigations were often provoked by direct clinical experience with ‘abnormal’ variants of certain conditions. In the case of diabetes, for instance, British interest was raised following the publication of an article in *The Lancet* during 1955. In it, the author purported to describe a clinically distinct ‘J-Type’ of the condition; a type in which patients were young, thin and in need of insulin to achieve metabolic control (like type 1 patients), but who were generally insensitive to insulin's action and who were not liable to ketosis without treatment (like type 2).^37^ This was not the first time that a specific ‘tropical’ variant of diabetes had been discussed in the British press.^38^ Unlike earlier in the century, though, this time two academic clinicians from the University College of the West Indies followed up this publication, undertaking further work into the clinical course and metabolic changes in local diabetic patients, in conjunction with the MRC-funded Tropical Metabolism Research Unit.^39^ Furthermore, this work helped to foster a significant debate over clinical classification in mainstream British journals during the 1950s and 1960s—a discussion revolving around concepts of tropical difference often at the heart of classical tropical medicine.^40^ This discussion even attracted clinicians based in Britain, who in light of the publication became interested in the potential lessons to be learned by investigating their own postcolonial populations in the UK. And, although initially sceptical, they too ultimately undermined claims to a specifically tropical diabetic variation.^41^

Evidence of common chronic diseases in populations deemed socially and biologically different to those ‘at home’ attracted British researchers, and ultimately prompted funding bodies to demonstrate great interest in colonial populations. The 1950s and 1960s were decades of significant change in the public health interests of British doctors, scientists and policy makers. Whilst interest in ‘chronic disease’ as a broadly-conceived object of policy did not really emerge until the early 1960s (and then only in a stuttering manner), British epidemiologists and social medicine academics had nonetheless become interested in the morbidity and mortality patterns of a range of non-infectious conditions.^42^ Receding rates of infectious disease mortality were important in this regard, even if not sufficient on their own to cause shifting attention.^43^ Important too, here, were methodological innovations in studying prevalence and causation, and shifts in the cultural expectations and boundaries of medicine in relation to old age.^44^ New means for establishing causative relationships in non-infectious diseases were mobilised as academic researchers and clinicians framed chronic diseases less in terms of unavoidable degeneration and more in terms of lifestyle factors and culture.^45^ In the case of heart disease, for instance, interwar scepticism over the robustness of mortality figures slowly gave way, as links to sugar and fat consumption emerged from British and international research during the 1940s and 1950s.^46^ Moreover, novel techniques for measuring the morbidity and disease burden in nationalised health and welfare structures also highlighted startling increases in linked conditions during the 1950s and 1960s, doubling estimated rates of diseases like diabetes mellitus.^47^

The changing focus of a leading MRC institution during this period, the Pneumoconiosis Research Unit (PRU), symbolises these shifts in both epidemiological and public health concerns.^48^ Initially interested in lung diseases of Welsh miners, by the early 1950s, the Unit's survey team had begun to move the research programme towards other common chronic conditions.^49^ Researchers applied the method of community surveys used to study lung disease and tuberculosis to the investigation of conditions like diabetes, hypertension and rheumatoid arthritis. Whole, well-defined populations of the general public were thus slowly enrolled in standardised diagnostic examinations, and although investigators had initially established comparative work as a means to standardise the measurements used, they also held comparison as the means to engage in preventive work. Once prevalence rates of a specific condition were established, the scientific team argued, ‘clues as to aetiology … would appear in two ways: (i) by establishing differences in prevalence of the same disease in different areas, or between different occupations in the same area; [and] (ii) by providing a complete unselected population of a particular disease group whose characteristics can then be compared with a proper control group’, free from the disease, and matched for key demographic characteristics.^50^ The difference in distribution of disease between populations, that is, would offer indications as to the hereditary, physiological and environmental factors generating disease, and thus offer opportunities for further research. Once such factors were elucidated, surveillance would need to be maintained to follow-up associations over time.^51^

With the existence of prominent non-infectious diseases noted overseas, British researchers in the 1950s were quick to seize on the potential power of comparison between its own populations and those of its colonies. An editorial piece in *The Lancet* expressed the logic and motivation for this move clearly during 1956. It argued that British funders and researchers should back ‘medical research in the Caribbean’ because it ‘may pay big and rapid dividends in the future’.^52^ ‘Help in the elucidation of diseases common in Britain’ the journal proposed, ‘may well come from a study of the differences they show in areas like the Caribbean’.^53^ In other words, difference, so long the historical technology of rule in the colonies, provided the lens through which British medical scientists read disease in colonial populations, but now for a very different purpose than government. Local clinical interests may have raised awareness of chronic diseases in colonial locations, but British researchers and funders framed their colonial engagements in terms of national benefit. They sought no role in the putative colonial enterprise of reshaping life and society in the colonies, that is, aside from making research subjects out of political subjects in the novel British fight against chronic disease.

In fact, the shifting interests of the PRU also provided a means through which an epidemiological interest in population contrast in Britain mapped onto the colonies. In the late 1950s, the two leads on the PRU community surveys—Archie Cochrane and Bill Miall—applied to the CMRC for funds ‘to determine whether the techniques [developed in Wales] could be applied in less developed areas, where the prevalence of [chronic] diseases was thought to be very different’.^54^ Buoyed by their initial results, these researchers gained grants for further studies in 1958–59, and applied to establish a new unit to ‘measure the prevalence and attack rate of a number of common diseasese’ and to ‘make a series of comparative studies between Jamaica and Wales’.^55^ Although focusing particularly on cardiovascular disease, Cochrane and Miall argued that their work would offer insight into the distribution and potential causes of a number of major chronic diseases, and would thus ‘exploit the opportunities for … research [in Jamaica] and in the Caribbean generally on a larger scale’.^56^

These potential opportunities for medical research in the Caribbean, moreover, inextricably linked epidemiological interests with imperial politics. First, Miall and Cochrane saw utility in the racial difference of the targeted population, and the social conditions prevalent across the British Caribbean. As elsewhere in the Empire, race played a central role in structuring political and economic life in the Caribbean colonies, and the structural legacies of slavery continued to cast long environmental, social and cultural shadows.^57^ Medicine had provided a prominent domain for articulating constructs of racial difference, and whilst racialised discourses often focused on ‘unhygienic’ social practices, biological otherness nonetheless continued to provide a central investigative frame for mid-twentieth century researchers.^58^ The ‘travellers’ tales … of a high prevalence of hypertension, rare ischaemic heart disease and atypical diabetes mellitus' were of interest, Miall and Cochrane recalled, as Jamaica's assumed biological and social difference provided them with an opportunity to profitably compare distribution and causation with Welsh communities.^59^ British interest in the interactions of biology and society in the production of disease thus mapped neatly onto these colonial structures.

Secondly, as epidemiologists, Cochrane and Miall thought longitudinally, and sought to link their work to colonial and post-colonial projects of modernisation. British projects for colonial development had stoked interest in theories of modernisation during the 1940s and 1950s, whilst decolonisation and the Cold War made the object of development the concern of independent states and international agencies.^60^ Echoing earlier colonial practice, post-war policy makers and development agencies often saw medicine and public health programmes as central to the process of development. Such programmes, they believed, would both modernise the citizenry and remove health barriers to productive labour and economic change.^61^ Cochrane and Miall, however, sought to turn development's concomitant transformations in social environment into a research opportunity. The Caribbean, they believed, sat on the threshold of modernisation, and the new unit would allow them to investigate the ‘influence of the expected rapid change in living conditions on the pattern of disease’. In other words, to map epidemiology in conjunction with economic change.^62^ The importance of such changing conditions to disease profiles could not be studied within Britain's own already-developed environs. They could, however, be studied in the colonies, helping to disentangle the importance of certain cultural behaviours, forms of socioeconomic organisation, and specific physiological markers to the onset of chronic diseases over time. Via concepts of development, that is, epidemiological interest aligned neatly with colonial politics and its legacies.

Finally, Cochrane and Miall also consciously tied medical opportunities to the administrative machineries and political ties forged through colonisation. As Cochrane and Miall put it in their application for a unit, Jamaica had been chosen for two reasons. First, because it had a ‘predominantly English-speaking Negro population whose age statements could be checked’ and, secondly, because even in the face of expected independence, researchers would be able to assume responsibility for health care provision in specified areas.^63^ Furthermore, in terms of planned work for the 1960s, British medical scientists had already made links with communities of interest in collaboration with existing research institutions in the area. Cooperation, therefore, could be easily resumed upon the opening of the Unit. Researchers thus valued Jamaican communities, both because they could be investigated through a recently established cultural and institutional research infrastructure, and because populations could accessed, communicated with, and registered in a survey's intense bureaucratic monitoring system.^64^ Both were products of British colonial projects.

Couched in a framework of utility and opportunity, Cochrane and Miall's application proved persuasive to the Committee, and to its successor the Tropical Medicine Research Board (TMRB).^65^ After finishing up work remaining in South Wales, Miall returned to Jamaica in 1962 to assume his position as Director of the new Epidemiological Research Unit (ERU), Jamaica—an institute that the MRC explicitly discussed as a collaborator for a twin institution in Cardiff.^66^ Developments in Britain may have prompted the initial interest in chronic disease research, but it was colonialism that offered the conditions in which these communities could be seen as medically desirable, and subsequently be rendered studiable. And as the next section will explore, institutions like the ERU and researchers like Miall provided the means through which colonialism in turn left its mark on British biomedicine.

## The ERU, Hypertension and CHD: Researching and Preventing Chronic Disease Post-decolonisation

The end of colonial rule did not dampen epidemiological interest in the power of difference for elucidating causes of chronic diseases, and British funding continued to concentrate in former colonial territories. Although only a recent and comparatively minor part of British overseas funding, British chronic disease research continued to operate with a colonial edge beyond the end of imperialism.^67^

There were, of course, significant political and infrastructure changes which institutions like the ERU had to negotiate. Although the multiple agencies of the colonial state were determined to keep the Empire together during the 1940s and 1950s, such an aim was made impossible by economic difficulties, anti-colonial movements, and a changing international environment.^68^ Between 1957 and 1965, the vast majority of the empire was dissolved, with the most significant colonies for researchers achieving statehood.^69^

The creation of independent governments was also accompanied by international organisations intensifying their role in medical research and policy-formation networks in former colonial territories.^70^ To be sure, this was not a novel development. International agencies and private philanthropies had penetrated colonial boundaries throughout the twentieth century, and economic and political links between the Caribbean and the USA had also manifested in American involvement in pan-imperial institutions.^71^ Nonetheless, interaction between former colonies and bodies like the World Health Organisation intensified after decolonisation, in ways that would influence British interaction with newly-independent states.

Finally, change was also felt in Britain itself. In terms of chronic disease research, the Tropical Medical Research Board replaced the CMRC as the key coordinating body for state-funded research overseas.^72^ The Board had been created in 1960, following recognition in the Colonial Office and the CMRC that a forthcoming wave of colonial independence would make important sites of research ineligible for receipt of CD&W funds.^73^ Fear of losing valuable research institutions convinced both the Colonial Office and the MRC that change was required. The composition of the Board remained very similar to the previous Committee, and it assumed responsibility for the institutions previously funded through the CMRC.^74^ However, the TMRB was brought into the Medical Research Council's administrative structure, so whilst it continued to advise the MRC and the government departments responsible for Commonwealth relations, the Council provided the framework for its decision making. The change meant that the Board's interests were now subject to the MRC's changing priorities and financial fortunes to a far greater extent than before and, as noted later, this would have important results during the financially straightened years of the 1970s.

Opening on the eve of Jamaican independence, the ERU moved across both the colonial and emergent post-colonial order. On the one hand, its creation and construction owed much to colonial structures. The MRC and Wellcome Trust provided funding for its capital infratructure and early research programme, and it remained an MRC Unit in terms of its administration.^75^ On the other hand, however, throughout the 1960s and into the early 1970s, it received funds from international organisations like the WHO and, as the application for the Unit made clear, researchers were aware of the need to take into account the wishes of the newly independent government to garner support for its activities.^76^ In exchange for the Government of Jamaica allowing the Unit to monopolise health care for its chosen communities, for instance, the Unit undertook operational research to help assist state efforts elsewhere.^77^

Yet, the core of the Unit's research programme in the decade following its opening remained deeply connected to the British debates that had powered its launch. This was perhaps most visible in relation to its work around hypertension and arterial pressure. The Unit's work in this regard concerned a long-term prospective study of arterial pressure in the defined rural community of Lawrence Tavern, a village occupying a ‘rugged inland area’ 20 miles from Kingston.^78^ With the initial 1959 surveys providing the baseline data, the population was used for a number of research purposes, most of which related to cardiovascular disease.^79^ The hypertension work, however, had initially been oriented towards intervention in the debate about essential hypertension emerging in Britain between George (later Sir George) W. Pickering and Robert Platt during the 1950s.

The basis of the debate has been explored thoroughly elsewhere.^80^ In short, Platt, through his studies of clinical populations with malignant hypertensive disease, had argued that essential hypertension was a discrete disease entity. Assessing his patients and their relatives, he proposed that the condition occurred bimodally as the result of a largely Mendelian inheritance, ensuring that individuals either suffered from the condition or did not. Pickering, by contrast, surveyed blood pressure in non-hypertensive hospital populations, and framed blood pressure as a continuously distributed variable. Hypertensive disease for him occurred at the upper end of the scale. According to Pickering, therefore, the aetiology of the condition was a mixture of polygenic inheritance and environmental influence, and significant debate emerged in relation to explaining the quantitative relationships found, particularly between relatives.^81^

Miall's entrance into this exchange began whilst at the PRU. Both he and Cochrane had previously worked with Pickering. Miall had worked briefly as Pickering's House Physician at St Mary's Hospital during 1950, whilst Cochrane had been Pickering's collaborator as part of a team at the University College Hospital; a team which included such present and future luminaries in British medicine as Sir Thomas Lewis (head of the renowned Clinical Research Department at UCH), Harold Himsworth (later Sir Harold Himsworth, Secretary of the MRC from 1949 to 1968, and Chair of both the CMRC and TMRB during those years), and Philip D'Arcy Hart (who, along with Pickering, would work with Miall and Cochrane on their research at the PRU).^82^ Miall in particular had shared Pickering's interest in blood pressure, and sought to use his connections with the Welsh communities to engage in the debates about hypertension through longitidunal studies of ‘normal’ (i.e. non-clinical) populations. Such research assumed even greater significance once surveys like Miall's had linked certain levels of blood pressure with cardiovascular disease—one of the major concerns of Britain's new public health.^83^

By 1959, Miall had come to agree with Pickering that hypertension was a quantitative abnormality of arterial pressure, and that it was polygenically inherited. He had, however, also noted several possible influences on arterial pressure in the population, including age, occupation, salt intake, and parity (for both men and women).^84^ This work was extended into Jamaica, not just as a means to standardise research measures for arterial pressure, but also to test his theories in a population with different social and biological compositions. As noted in Miall's first co-authored publication from this work, research in the USA and the West Indies had suggested that ‘the arterial pressure in negro populations is higher than that for white populations’, but ‘the relative contributions of environment and genetics have still to be defined’.^85^ As tests for arterial pressure could vary between surveys and observers, Miall used the same staff and examination techniques as in Wales. In doing so, he hoped to rule out potential biases when ‘determin[ing] the magnitude of differences in blood pressure between negroes in Jamaica and whites in South Wales’ and thus be best placed to ‘determine whether such racial differences in arterial pressure as might be found were explicable in terms of difference in the nature or magnitude of environmental or genetic factors’.^86^

Although discussing ‘environmental’ causes, Miall and his team considered these broadly, taking in housing conditions, diet, employment type and income, as much as climate and terrain. The survey tried to ‘match’ these factors in rural and urban Jamaicans, although with some difficulty.^87^ Nevertheless, Miall concluded that the research in Jamaica served to support many of the findings from the Welsh survey.^88^ Parity and age both appeared to influence pressure to some degree, and average regression scores for relatives matched those found in the Welsh populations, thereby ‘add[ing] further weight to the evidence that arterial pressure, if inherited at all and not merely similarly influenced within families by common environmental factors, is polygenically determined’.^89^ Despite supposed racial difference, that is, Miall concluded that common genetic or environmental factors were most likely to influence blood pressure as a universal principle.

That the article appeared in the prestigious *British Medical Journal* (*BMJ*) was not just a reflection of the periodical's broader interest in chronic disease research in tropical locations.^90^ It also symbolised Miall's grounding in British debates about arterial pressure and hypertension. Indeed, he and his colleagues had targeted the *BMJ* previously, and continued to target it as a site for important work arising from the Welsh communities.^91^ The authors' decision to aim for the *Journal*, moreover, also earned the article a broad domestic and international audience. Researchers in Britain, the USA and other former colonial locations referenced the Jamaican research in their own work, including it in discussions about the importance of social and genetic factors in blood pressure levels.^92^ Along with rural–urban comparisons in Britain, these investigators referred to Miall's team's finding that rural Jamaicans had higher mean blood pressure than their urban counterparts, and followed Miall and his colleagues in comparing American and African research with the Jamaican work. They also drew the same conclusions about the importance of age, and common genetic and social influences on blood pressure, here providing a means through which knowledge returned to the colonies to influence domestic debates.^93^

In later work, published before his return to the UK in 1971, Miall seemed to change his opinion on certain aspects of blood pressure. Research in Pacific Island populations (with Australian funding, but also some support from the Nuffield Foundation), had indicated that pressure did not always increase with age, giving greater potential significance to social and environmental influences.^94^ By contrast, maintaining connections with the ERU in South Wales, further analysis of the Welsh data lead Miall to suggest that there might be some self-propelling factor at play in the rising pressures over time. Accepting the Pacific Island conclusions that increases were not always inevitable, Miall instead suggested that over a certain threshold, increases in pressure were proportional to the base finding. That is to say, though still finding some role for environmental factors, Miall proposed that over the same period of time, individuals with higher pressures would experience greater increases in pressure than did individuals with lower pressure.^95^

Follow-up work from Jamaica appeared to disprove this theory and reassert modifiable factors underpinning pressure levels. Rather than the expected changes, this research indicated that systolic pressures increased less than anticipated, whilst diastolic pressures actually fell over time.^96^ And although Miall's team initially tried to write off this deviation as an artefact of survey methods and treatment, the puzzling connection between time, age and environment persisted in discussions of hypertension.^97^ Indeed, Miall was careful about drawing causal relationships from associative trends, and for him, as for others involved in hypertension research in the post-colonies, the challenge remained to locate the threshold at which pathology began, and to find the triggers involved.^98^ Research into non-Western populations would thus continue to find importance here.

Transmitted through lectures and collaborative research projects, the later ERU research into cardiovascular disease more broadly was also of interest to British and international organisations. In 1972, for example, the internationally renowned cardiovascular epidemiologist, A. G. Shaper, delivered the prestigious Milroy Lectures at the Royal College of Physicians of London. Shaper had qualified in Cape Town, but had been in receipt of MRC and British state funding since the 1950s, working in Uganda until 1970, and then in various London institutions until retirement in 1992.^99^ His lectures were grouped under the title ‘Cardiovascular Disease in the Tropics’, but Shaper was explicit that ‘the differences in natural history made evident from tropical experience give a perspective on disease in our own community that we cannot possibly obtain from studies limited to our own environment’.^100^ Research not just from Miall's Unit, but from various international sources, was used to make the case for the importance of environmental and social causes of cardiovascular disease, particularly in the instance of hypertension.^101^

Through connections to the elite of British epidemiological and general practice research, moreover, Miall's and Shaper's claims were repeated in large-scale review literatures, such as Julian Tudor-Hart's 33-page review on managing hypertension in general practice in 1975.^102^ As another graduate of the south Wales Pneumoconiosis and Epidemiological Research Units, Hart would have been familiar with Miall's work, and used it along with Shaper's to discuss the importance of parity, salt intake, age, genetics and previous blood pressure when assessing patients.^103^ Beyond this, as a well-respected general practitioner and researcher, Hart worked in influential institutions in British medicine, bringing this broad knowledge of arterial disease, for instance, to Royal College of General Practice advisory bodies for preventive health.^104^

Indeed, the colonial impact on British medicine may also have been carried over in less obvious ways. Both Miall and Shaper returned to the UK in the 1970s, and both went on to carry out further work in cardiovascular disease in British MRC institutions. After 10 years in Jamaica, Miall found employment at a new MRC institution at Northwick Park. Whilst there, he served as Scientific Secretary to an expansive and incredibly influential MRC trial on the treatment of mild hypertension, based in general practice.^105^ Whilst the origins of the trial lay far beyond Miall himself—in the politics of the MRC, as well as in a mixture of commercial practices, scientific research, and clinical experiences—colleagues in Miall's Jamaican Unit had interestingly undertaken much smaller therapeutic trials in mild hypertension during the 1960s.^106^ Similarly, Shaper's continued linking of individual and environmental risk factors in cardiovascular disease on his return to the UK also hints at the enduring influence of his colonial experience.^107^ He became involved in the MRC-funded British Regional Heart Study, and published important work around high-density lipoprotein (HDL) cholesterol. Crucially, though, low HDL cholesterol was at that point in time considered a potential risk factor for ischaemic heart disease in light of work by another PRU graduate with Caribbean research experience, George James Miller.^108^ The continued entanglement of post-colonial and British research institutions and questions into the 1970s, then, reflected the deep connections between metropole and colony forged during the late colonial interest in chronic disease of the 1950s. And researchers like Shaper and Miall were able to rise to the tops of their profession through colonial and post-colonial experiences that had been predicated upon these colonial interests and research frameworks.

## Conclusion

Miall's departure from the ERU threw the Unit's future into question. The Board had known for some time that Miall did not intend to stay in Jamaica indefinitely, and had began seeking replacements two years prior to his leaving.^109^ In fact, concern about the potential loss of another research site led Board members to consider opening a new Unit in the Caribbean. Evoking a sense of old imperial strategy and geopolitics, they argued that political instability elsewhere in the tropics had reduced potential sites of British access, whilst experience with institutions in East Africa had demonstrated that ‘indirect control may become dangerously ineffective’.^110^ Establishing a new Unit, it was proposed, would not only provide ‘an important centre of medical research in the tropics’ but also ‘give the Council an overseas base in a shrinking world’, an ‘extra insurance against possible deterioration of the present’.^111^ Ultimately, the old Rockefeller laboratory at the centre of the Board's plans was taken over by the Pan-American Health Organisation and remade as the Caribbean Epidemiology Centre.^112^ The Board did find a new Director for the ERU, but he left after becoming sceptical about the value of further chronic disease research in the Caribbean.^113^ Having invested so much in the Unit, though, the Council decided to reformulate it as the MRC Laboratories (Jamaica) (on the lines of another institute in the Gambia), and it redirected research towards sickle cell anemia—another disease of interest to post-colonial Britain.^114^

Cardiovascular disease and chronic disease epidemiology did not disappear from the agenda completely, however. The MRC supported its scientific staff to work at internationally funded institutions on shorter-term programmes. As noted, George Miller worked at PAHO's Caribbean Epidemiology Centre, during the second half of the 1970s. And Miller himself was central to establishing an important longitudinal study in Trinidad—the St James' Heart Study—run in collaboration with the US Centres for Disease Control and Prevention, as well as with the WHO. Furthermore, it was a study which received funding from a broad range of actors, including PAHO and the Government of Trinidad as well as the MRC.^115^ In fact, the study was not the only large-scale enterprise in this mode, with other work in this direction carried out in East African territories.^116^

The involvement of the WHO and national government departments in this latter research of course raises important questions about the coloniality of the work undertaken after the end of empire. In Trinidad, as elsewhere, international institutions like the WHO worked with independent states—sometimes at the invitation of the latter—whilst they also played vital roles in the publication of research results.^117^ Moreover, British research in former colonial locations would also appear in major international periodicals. For example, alongside publishing in *The Lancet* and the *Bulletin of the World Health Organisation,* the teams from the Epidemiological Research Unit also published the results of follow-up studies in US-based journals with transnational readerships, like the *Journal of Chronic Diseases*.^118^ Likewise, figures like Miall engaged with WHO expert committees and scientific conferences, taking part in networks beyond nation and empire.^119^

Given the way in which British medical scientists assumed roles in networks of exchange on various scales, and in light of how research in post-colonial locations was referenced far beyond its initial site of production, does this mean that British research should be read in terms of other conceptual frameworks? Perhaps as forms of international or global medicine? I would suggest not. As has recently been emphasised by Sarah Hodges and Warwick Anderson, colonial structures underpinned both the careers of the researchers discussed here, and the manner in which their research was conducted, during and after the end of British imperial rule.^120^ On the one hand, researchers engaging in international networks were able to do so precisely because their colonial careers gave them recognised expertise with conditions and populations in former colonial locations. The move from colonial medical to international health structures was, of course, not unusual, and even in instances where researchers moved from one site to another, continued colonial links could be maintained.^121^ Miall, for instance, was invited to participate in a conference in New Zealand towards the end of his Jamaican stay, to ‘advise the WHO on the potential use of the migration going on from the Polynesian islands into New Zealand in assessing the effects of the changes in the environment on cardiovascular disease’.^122^ Here, then, he swapped former colony for former dominion.

On the other hand, research structures themselves retained their colonial shape. British research continued to flow to and from strategic points in the post-colonial world, largely determined by where advantageous relationships could be maintained after empire. These links provided a means for the post-war epidemiological interest in the power of difference to continue to map onto colonial geographies beyond colonialism. Research continued to be performed on populations and disease profiles that provided an ‘other’ for the British population. Opportunities and a sense of utility in post-colonial visions were thus persistently tied to these interests, and knowledge returned to Britain in exchange.

Of course, state institutions had played a significant role in making bodies useful ‘at home’ in the twentieth century, and trials themselves could be seen as a colonising part of biomedicine.^123^ Yet, the colonial nature of British encounters in the previous colonial territories can be seen in the way that actors used British national interest and colonial difference to justify and encourage research, as well as in the power relations that framed such engagements. In both the questions asked and the knowledge gained, a colonial heritage thus left an indelible mark on supposedly metropolitan knowledge. And this colonial edge was defended at the very heart of the British scientific infrastructure by major figures like J. N. Morris, who argued during an important MRC policy review in the 1970s that:

Since the end of the last war increasing attention has been given to the study of non-infectious diseases in the tropics. It quickly became apparent that there are remarkable differences between tropical and temperate countries, and within the tropics, in the pattern and distribution of some diseases. Analysis of such differences provides an important opportunity for a better understanding of the aetiology of these diseases, and may therefore bring direct benefits to medicine in the UK.^124^

To be sure, the outcomes of colonial research for UK medicine varied, particularly where ideas of race and ethnicity in medicine were concerned. Although interested in both heredity and environment, the question of race framed much of the research across international and post-colonial networks. Comparisons were not always between a supposedly developed white British population and an homogenised ethnic other. Foreshadowing contemporary practice, for instance, the ERU team used black populations in the colonies as points of comparison for others in different parts of the world. Thus black populations in the Caribbean were compared with African American groups in the USA, as well as with communities in West Africa, primarily on the grounds that researchers presumed they would all share a genetic heritage.^125^

As Roberta Bivins has recently argued, when tropical populations were transplanted to Britain, this interest in biological population differences became the grounds for discussing pathology.^126^ Despite a long tradition of migration, changes in the body politic of Britain became significantly more visible during the close of Empire.^127^ Initially, migrant communities were discussed in medical—and political—terms in relation to various epidemics of diseases that Britain had allegedly conquered in decades past.^128^ By the 1970s and 1980s, however, doctors could no longer ignore the presence of black and Asian populations in British chronic disease clinics, and clinicians organised prevalence surveys and research programmes with the aim of determining resource implications for the NHS.^129^ Emergent from this work, however, was a distinct interest in ethnic differences in ‘susceptibility’ (rather than in universal risk factors), and discourses of race and ethnicity drew upon research performed in colonial and post-colonial locations as sources of comparison once more.^130^ Indeed, as in the research and care of other conditions, a number of the doctors involved in such work had previous colonial and overseas experiences, and brought back their networks and interests with them.^131^

Yet, as the history of British cardiovascular disease epidemiology would suggest, it is clear that comparisons between supposedly similar ‘racial’—later ethnic—groups also provided researchers with the means to compare the affects of environment and social structure on disease.^132^ That is, to find potentially universal causes for Britain's ‘modern epidemics’, and thus to influence British medicine for the whole population. Whilst not a central focus of post-war state-funded research in colonial and former colonial locations, chronic disease research in these sites undoubtedly left a mark on the research practices and knowledge base of British medicine. It is a history which historians of colonial and metropolitan medicine would greatly benefit from exploring further, and which historians of Britain's own ‘new public health’ could integrate much more convincingly into existing, non-hierarchical narratives of national and international medical change.
